# 51727 A Systematic Review of Implementation Science Frameworks Used in Cancer Prevention Interventions

**DOI:** 10.1017/cts.2021.744

**Published:** 2021-03-30

**Authors:** Serena Xiong, Hamdi Abdi, Rebekah Pratt

**Affiliations:** 1 University of Minnesota School of Public Health; 2 University of Minnesota Medical School

## Abstract

ABSTRACT IMPACT: Specific recommendations will be suggested in this presentation as to how a health equity lens can be applied to implementation science frameworks. OBJECTIVES/GOALS: This systematic review consolidated literature on how implementation science (IS) frameworks (e.g., RE-AIM) have been used in cancer prevention services (e.g., screening, tobacco cessation programs) to reduce health disparities. METHODS/STUDY POPULATION: The systematic review was conducted in accordance with PRISMA and registered with PROSPERO. Searches were conducted in Ovid MEDLINE, PubMed, PsycINFO, CINAHL, and EMBASE between January-May 2020. Search strategies used the combinations of terms related to implementation science frameworks, cancer prevention and/or intervention, and all search algorithms were validated by a public health librarian. RESULTS/ANTICIPATED RESULTS: A total of 1,025 articles were screened and 84 were deemed eligible for full-text screening. After full-text screening, n=27 articles were included for data abstraction and synthesis. Of the 27 studies that used an implementation science framework, only one-third of studies (N=9, 33.3%) used an IS framework to address cancer-related health disparities. Of those nine studies, six of them used the Consolidated Framework for Implementation Research (CFIR) to guide, inform, and/or adapt the implementation of a cancer prevention intervention to target health disparities. However, the variability in how this framework was applied remains a challenge. DISCUSSION/SIGNIFICANCE OF FINDINGS: Recommendations for how various IS frameworks can be used to address cancer prevention disparities will be presented, such as, guiding principles on how to intentionally select domains within the CFIR that will capture input from key stakeholders in health disparities populations.

